# LAILAPS: The Plant Science Search Engine

**DOI:** 10.1093/pcp/pcu185

**Published:** 2014-12-04

**Authors:** Maria Esch, Jinbo Chen, Christian Colmsee, Matthias Klapperstück, Eva Grafahrend-Belau, Uwe Scholz, Matthias Lange

**Affiliations:** Leibniz Institute of Plant Genetics and Crop Plant Research (IPK) Gatersleben, Corrensstr. 3, D-06466 Stadt Seeland, Germany

**Keywords:** Functional gene annotation, Information retrieval, Integrative search engine, Plant genomics resources, Traits

## Abstract

With the number of sequenced plant genomes growing, the number of predicted genes and functional annotations is also increasing. The association between genes and phenotypic traits is currently of great interest. Unfortunately, the information available today is widely scattered over a number of different databases. Information retrieval (IR) has become an all-encompassing bioinformatics methodology for extracting knowledge from complex, heterogeneous and distributed databases, and therefore can be a useful tool for obtaining a comprehensive view of plant genomics, from genes to traits. Here we describe LAILAPS (http://lailaps.ipk-gatersleben.de), an IR system designed to link plant genomic data in the context of phenotypic attributes for a detailed forward genetic research. LAILAPS comprises around 65 million indexed documents, encompassing >13 major life science databases with around 80 million links to plant genomic resources. The LAILAPS search engine allows fuzzy querying for candidate genes linked to specific traits over a loosely integrated system of indexed and interlinked genome databases. Query assistance and an evidence-based annotation system enable time-efficient and comprehensive information retrieval. An artificial neural network incorporating user feedback and behavior tracking allows relevance sorting of results. We fully describe LAILAPS’s functionality and capabilities by comparing this system’s performance with other widely used systems and by reporting both a validation in maize and a knowledge discovery use-case focusing on candidate genes in barley.

## Introduction

Modern molecular biology encompasses a broad range of methodologies, ranging from experimental data acquisition on genes and proteins to post-genomics technologies, such as RNA sequencing, phenotyping, proteomics, systems biology and integrative bioinformatics (Kitano 2002). With the current wave of new and cheap technologies, vast amounts of data are being generated at an unprecedented rate (Schadt et al. 2010). As a consequence, the number of annotated and functionally analyzed plant genomes, and publications of these genomes and gene products, is also on the rise. In September 2014, the UniProt protein knowledge base had over 82.6 million entries (UniProt Release Statistics 2014, http://www.uniprot.org/statistics/). Additionally, the NCBI GenBank plant division provides access to around 25 million sequences (NCBI Nucleotide Plant Division Statistics 2014, http://www.ncbi.nlm.nih.gov/nuccore?term=%22pln%22[Division]) and PubMed comprises >24 million citations for biomedical literature from MEDLINE, life science journals and online books (NCBI Nucleotide PubMed Statistics 2014, http://www.ncbi.nlm.nih.gov/pubmed?term=%2201%2F01%2F0000%22%5BPDAT%5D+%3A+%223000%22%5BPDAT%5D). Many information systems are specified in different broad subareas; for example, Gene Ontology (GO; Ashburner et al. 2000) and Plant Ontology (Cooper et al. 2013) are two ontology information systems. Furthermore, a number of individual platforms for different organisms, such as the Arabidopsis information resource (TAIR; Lamesch et al. 2011) or RAP-DB, the Rice Annotation Project Database (Sakai et al. 2013), have been developed. Overall, >1,552 life science databases are publicly available (Fernández-Suárez et al. 2014).

Despite this enormous amount of publicly available information, the search for candidate genes and relevant genomic data is a time-consuming and sophisticated task (Divoli et al. 2008). In recent years, information-processing methods have evolved from library research and individual data archives to web-based systems, cloud-computing and distributed databases. To manage the rapidly increasing amount of big, complex data, database information systems are increasingly leveraged (Stein 2010, Lange et al. 2014). This increased usage has resulted in a real need for improved information retrieval (IR) methods.

Typically, scientists make rather vague queries because their searches are often explorative with no clear expectation of the results. These vague searches pose a problem for current databases and information systems, as queries of this type cannot be semantically interpreted without comprehensive semantic document tagging or the use of controlled vocabulary (Merelli et al. 2014). Further underlying issues include data distribution and isolation, structural heterogeneity, missing metadata and query languages. It is becoming necessary to rank query results in an intuitive way that fulfills the information needs of an individual researcher, a process that requires suited IR methods such as statistical (e.g. TF–IDF, short for term frequency–inverse document frequency) or probabilistic relevance algorithms, vector space models (VSMs) or PageRank, which is used by Google. TF–IDF is a statistical method that shows the importance of words to a document in a corpus. Probabilistic algorithms aim to estimate the relevance of a document to the given query, whereas the VSM takes documents as vectors, comparing the angles of each document. PageRank measures the number and quality of page links, where the importance of a web page rises with the number of links to said web page. A selection of available literature resources for biomedical research and the underlying methods of these systems are summarized in Kim et al. (2008).

A well-known and frequently applied system for IR is Google. It works well for general information but has deficits in more specific information searches such as the retrieval of candidate genes. More dedicated life science search engines and information systems for gene annotations are available. As big data and the difficulties associated with analyzing and querying such data increase, the number of life sciences IR systems is also increasing. Many of these published IR systems are based on the Apache Lucene or BioMart frameworks. Comprehensive platforms for integrative database searches, such as NCBI GQuery (NCBI Resource Coordinators 2014) or IntegromeDB (Baitaluk et al. 2012), have been developed. The number of databases available in GQuery is extensive, but the restriction to navigate through each database separately, as opposed to performing an extensive cross-database search, is a time-consuming task. Conversely, IntegromeDB applies state of the art IR technology to scan heterogeneous, multidomain web resources and databases for >1,000 organisms and compiles comprehensive knowledge reports. UniProt (UniProt Consortium 2014) is a popular resource for protein sequences and functional annotations, with both reviewed (UniProtKB/Swiss-Prot) and unreviewed (UniProtKB/TrEMBL) sequences available to the user. The user is able to perform differentiated searches. Results are sorted by the UniProt default score or by defined fields. Ensembl Plants (http://plants.ensembl.org/) stores genome information for different plant species, though the search options are less comprehensive than those available in UniProt. Gene information gets visualized and sequence data can be downloaded by the user. More general systems that integrate different data sources are EB-eye (Valentin et al. 2010), DBGET Search (Fujibuchi et al. 1998), GO (Ashburner et al. 2000) and MIPS PlantsDB (Nussbaumer et al. 2013). Moreover, PubMed (NCBI Resource Coordinators 2014) comprises citation abstracts from life science journals and is part of the search engine GQuery.

The above considerations, an extensive literature study, daily work with public IR systems and personal experience have motivated us to compile a catalog of minimal requirements for IR systems in the frame of plant genome research. Based on our experiences, we feel that the most important aspects for an efficient and user-friendly IR environment in life science are described as follows.

In terms of technology, it is important to integrate a non-replicated set of miscellaneous data domains to offer a compact, comprehensive information source. To achieve the best results, the data should be based on the latest available facts and data sources (age of data). In terms of information depth, the system must be able to extract all available cross-linked data stored in the documents to deliver a high degree of structural and interconnected information (data range). Furthermore, a result-ranking and filtering mechanism is useful to define and collect relevant information (ranking). There are several criteria influencing the user’s decision to use a certain result; therefore, essential components of an IR system include personal user profiles delivering results with high personal relevance (pertinence) and integration into downstream workflows (data cart).

With these requirements in mind, we have developed a tailored IR system that allows efficient querying of plant genomic resources.

## Results

**Fig. 1:**
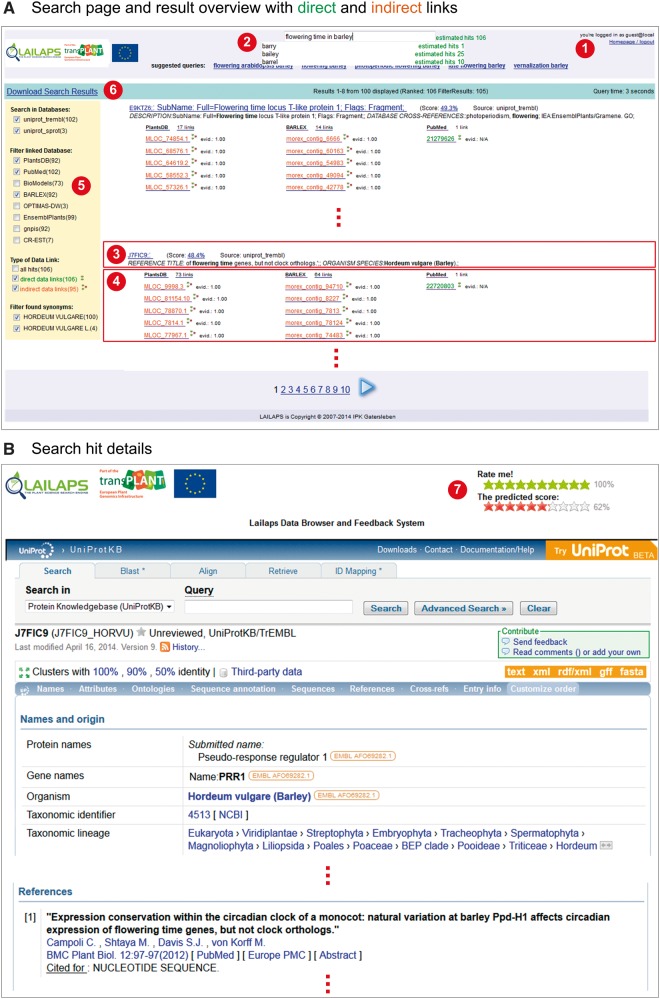
Screenshots of the LAILAPS web interface: (A) illustrates the search and results page, where the user logs in (1) and starts the search with a request (2). Spelling correction and an estimation of the expected results are shown. All hits, including a short excerpt of relevant text positions (3) and a list of annotation links to related information (4), are provided. Links can be direct (green) or indirect (red). Special filter options such as data sources or synonyms are located on the left side of the result page (5). All results can be downloaded as a Microsoft Excel sheet (6). Document links open a new tabulator (B) showing the database entry and a rating system (7) where the user can validate the obtained results.

Here we present LAILAPS (http://lailaps.ipk-gatersleben.de), a comprehensive IR system for exploring plant genomic data in a phenotypic perspective to support forward genetics research. LAILAPS’s focus is to support highly specific phenotype–genotype association studies. The system utilizes materialized integration of major information hubs in plant genomics, linked integration of specialized genome resources and effective IR technologies. LAILAPS applies established web search engine design patterns. Users can enter a keyword query and, when possible, are presented with available search alternatives and spelling corrections in addition to a number of expected results. The results are shown in order of relevance with excerpts from matched records and a list of cross-references to genome features. The user can download a complete result set containing all relevant information as presented in the result view as a Microsoft Excel file. User feedback is incorporated through an interactive, personalized rating system for each hit. Fig. 1 illustrates the major frontend components and the usability of LAILAPS.

We carried out a survey about frequently used life sciences databases among plant genome scientists from the transPLANT project (http://transplantdb.eu). As a result LAILAPS data source on 12 major indexed genome annotation repositories with a reference to 13 genome databases (see Table 1). To compromise between the need for short update intervals and the maintenance effort involved in these updates, the text index of genome annotation repositories is refreshed quarterly. Furthermore, novel genome annotations are published as a result of collaborative work and mid-term projects, and will be updated as references to genome databases on demand (the individual data updates are available at LAILAPS). Together, these methodologies ensure that LAILAPS is utilizing the latest available facts and resources, fulfilling our first requirement for an efficient IR system.

**Table 1:** Number of data entries for indexed and linked databases

Indexed database	Records	Linked database	Linked IDs
garlic shallot core collection	176	BMRF4Arabidopsis*^a^*	110,788
gene_ontology	40,730	BMRF4Glycinemax*^a^*	637,480
genebank information system of the ipk gatersleben	146,420	BMRF4Medicagotruncatula*^a^*	418,129
gramene taxonomy ontology	58,585	BMRF4Oryzasativa*^a^*	907,405
ncbi taxonomy	1,139,973	BARLEX	75,257
pdb	98,894	BioModels	34,597
pfam	14,831	CR-EST	2,066,967
plant_ontology	1,691	EnsemblPlants*^b^*	8,549,929
taxonomic allium reference collection	3,871	PlantsDB	131,928
trait ontology	1,327	PolapgenDB	10
uniprot_sprot	542,782	PubMed	64,766,473
uniprot_trembl	54,247,468	ensembl	4,198,205
		gnpis*^c^*	1,007,056
		Metacrop (link to conversions)	981
		Metacrop (link to substance)	253
		Optimas-DW	916,911

*^a^* Bargsten et al. (2014).

*^b^* http://plants.ensembl.org/.

*^c^* Steinbach et al. (2013).

In addition to data coverage depth, data quality is also an important characteristic of IR systems. Rather than flooding the user with data, LAILAPS applies ranking and filtering methods to predict the most reasonable resulting data items and ranks them by relevance in respect to the information needs of the user, fulfilling the data-ranking requirement we imposed on an IR system. This implemented approach scores 11 properties (described in the Materials and Methods) of a database entry, estimating the relationship between the query and the selected data item as well as static features of the item itself. To rank data items by their relevance, an artificial neural network is used and trained for specific users or user groups. An initial ranking network was trained by a molecular biologist with a strong background in systems biology and bioinformatics for an application in plant genomics. Furthermore, a feedback system enables the user to provide feedback that can be used to improve the prediction performance in general or to link individual trained neural networks with user profiles, ensuring the pertinence of this IR system.

Closely connected to IR specificity is the maximization of search sensitivity. LAILAPS utilizes a computer-aided query formulation that enables the system to include user expertise, facilitating the identification of data items that would not match ordinary keyword query string-matching algorithms. This is achieved by a real-time spelling correction (Esch et al. 2014), synonym expansion and the suggestion of related entries based on document similarity. To increase further the search sensitivity, the user interface supports advanced filter mechanisms for indexed databases, synonyms, additional genome resources and annotation evidence.

We compared the clarity and intuitiveness of the LAILAPS user interface with that of other widely used information systems such as UniProt, EB-eye, Ensembl Plants and others (a full list is found in Table 2). Most of these systems have an efficient data card mechanism as well as interactive data filtering and linkage. However, unlike LAILAPS, most systems do not provide a high personal relevance of queries, with only half of the systems ranking results. Query assistance is also a problem in most of these systems. Unlike LAILAPS, most systems provide either a query correction or a query suggestion. Therefore, in terms of pertinence, LAILAPS is superior to other available IR systems. A comprehensive summary of all criteria and systems can be found in Table 2.

**Table 2:** Summarized features of common plant search portals

	URL	Age of data	Data range	Ranking	Pertinence	Data cart	Query assistance	Interactive result filtering	Linking related data
LAILAPS	http://lailaps.ipk-gatersleben.de	Updated quarterly	Resources and annotations	Neural network	User Login and personalized search	Result download as Excel sheet	Query correction and suggestion	Filtering by data source	Linking to related data
EB-eye	http://www.ebi.ac.uk/ebisearch	Automatically updates and re-indexes data on a daily basis	Data resources hosted at the EMBL-EBI	Sort order is based on the proximity of the terms in the entries	Not provided	Possible via EMBL-EBI resource websites	Apache Lucene query syntax allowing query refinement through adding additional terms to the query	Result filter for sources	Explore related information
Google	https://www.google.com	Real-time upload	Entire web	Feature rank-based	Personalized search	Not provided	Query correction and suggestion	Filtering by many different criteria	Not provided
UniProt/UniProt Beta	http://www.uniprot.org/http://beta.uniprot.org	Updated and distributed every 4 weeks	UniProtKB, UniRef, UniParc, Supporting data	Sort by score (descending)	UniProt Beta provides a basket system	Data download possible in different formats	‘Did you mean’ function for small spelling errors in UniProt	Filtering by source	Cross-references
NCBI Entrez/GQuery (PubMed)	http://www.ncbi.nlm.nih.gov/gquery (http://www.ncbi.nlm.nih.gov/pubmed)	Depending on NCBI services (updated when new publications available)	All NCBI data	Sorting by relevance is possible	Login to NCBI provided	Different download formats provided	Automatic correction and query suggestion	Interactive result filtering is provided	Related citations in PubMed
Ensembl Plants	http://plants.ensembl.org	On demand	Genome information of different plants	Not provided	Personal configurations via login	Download of different file formats possible	Not provided	Filtering by species	External references
DBGET Search (Kegg)	http://www.genome.jp	Suited for maintaining large daily updated databases	Major databases: GenBank, EMBL, SWISS-PROT, PDB, PROSITE, EPD, PIR, PRF, KEGG Genes	Not provided	Not provided	Download RDF	Not provided	Not provided	Links to other DBs like UniProt, MIPS and more
IntegromeDB	http://www.integromedb.org	Re-loaded on a quarterly basis	http://www.integromedb.org/db-catalog.jsp	Relevance score	Not provided	Result download as CSV and RDF	Provides related suggestions	Not provided	Relations to query, synonyms and related information
AmiGO 2	http://amigo.geneontology.org/amigo	Built at regular intervals	Gene Ontology (GO) data	Alphabetical sorting	Not provided	Result table downloadable as txt file	Query suggestion	Interactive filtering by different criteria	Link to related internal data
MIPS PlantsDB	http://mips.helmholtz-muenchen.de/plant/genomes.jsp	Updated regularly, if new data available	Hosting of databases for different plant species	Not provided	Not provided	Genetic element download possible	No assistance provided	Automatically filtered by organism. No interactive filtering provided.	References provided

**Fig. 2:**
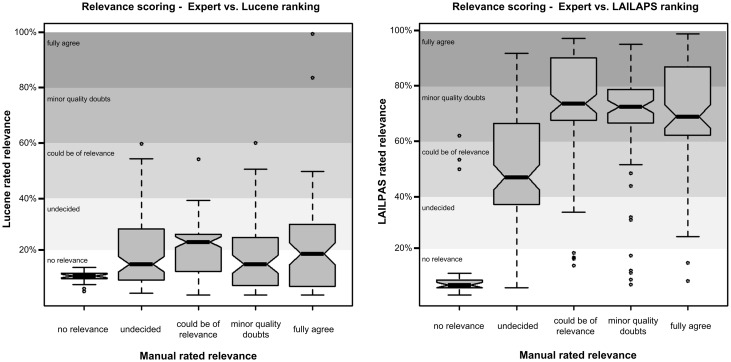
Comparison between the TF–IDF-based relevance ranking of Apache Lucene’s information retrieval API (left side) and LAILAPS neural network-based relevance prediction (right side). There are five classes of document relevance ranging from ‘no relevance’ to ‘fully agree’. A biological expert evaluated the relevance of a document. The boxplots show an improved ranking that separates relevant from non-relevant results using LAILAPS compared with Apache Lucene’s API.

To evaluate LAILAPS’s relevance ranking, a set of 20 IR query use-cases was selected (see Table 3) and ranked by the molecular biological domain expert. Result elements were randomly selected and classified into five relevance classes: ‘fully agree’; ‘minor quality doubts’; ‘could be of relevance’; ‘undecided’; and ‘no relevance’. The result of this evaluation is a set of 400 relevance-ranked database entries (Grafahrend-Belau et al. 2014). The evaluation results reveal that the LAILAPS ranking system effectively discriminates non-relevant and relevant results, but is less accurate for the classes ‘fully agree’ and ‘minor quality doubts’ (see Fig. 2).

**Table 3:** List of 20 traits and their expression as keyword queries

Query class	Subquery class	Query
Trait	Stress response	Salt stress
Trait	Agronomic traits	Yield
Trait	Morphological/ phenotypic traits	Ear emergence
Trait	Stress response	Barley salt stress
Trait	Agronomic traits	Barley yield
Biological entity	Protein name/ID	WUS protein
Biological entity	Gene name/ID	WUS
Biological entity	Gene name/ID	WUS Arabidopsis
Taxonomy	Cultivar name	Barley Morex
Taxonomy	Geography	Barley fertile crescent
Taxonomy	Subspecies name	Hordeum vulgare spontaneum seed
Aaffiliation	Institute name	MIPS muenchen
Affiliation	Institute name	Barley IPK
Metabolic function	Catalytic process	Sucrose synthase
Metabolic function	Primary metabolism	Photosynthesis barley leaf
Metabolic function	Metabolic engineering	Rice phytoene synthase
Metabolic function	Secondary metabolism	GABA barley
Regulatory function	Regulation of enzyme activity	Regulation of starch synthase activity
Regulatory function	Regulation of process	WUS regulation
Regulatory function	Regulation of process	WUS meristem

We further demonstrate the strengths of LAILAPS through the following two examples, which highlight the use of ranking algorithms and linked genome information.

### Example 1

To prove the functionality of our system, we chose an already known and published case linking information about candidate genes, contigs and protein analysis. This case encompasses a set of candidate genes in barley that are directly involved in flowering time and that influence the circadian rhythm, which plays a role in flowering time. Ariyadasa et al. (2014) recently published a map of cloned barley genes. One of the genes anchored on this map encodes Ppd-H1, a known regulator of the photoperiodic response in barley. To query LAILAPS, we chose two related keywords: (i) flowering time and (ii) circadian clock. Searching for ‘flowering time in barley’ resulted in over twice as many results as did ‘circadian clock in barley’, which is probably due to the more frequent use of the term flowering time. Nevertheless, both search results have in common that the known annotated gene MLOC_81154.10 is in the top 10 ranked documents (see Fig. 1). Therefore, these results support LAILAPS’s utility and accuracy in identifying plant genes and gene products of interest.

### Example 2

A typical IR use-case is that of knowledge discovery. A plant’s response to nutritional deficiency is an important agronomic trait; for example, the availability of inorganic nitrogen in the rhizosphere is a crucial factor governing growth rate and developmental patterning in higher plants (Takei et al. 2002). Therefore, it is important to study the expression and regulation of genes involved in inorganic nitrate metabolism, though the exact functions of all genes involved in this process have not been fully characterized. The following example demonstrates how a user can leverage LAILAPS to identify candidate genes and gene products for further characterization and validation.

LAILAPS includes references to OPTIMAS-DW, a comprehensive maize transcriptomics, metabolomics, ionomics, proteomics and phenomics data resource (Colmsee et al. 2012). Querying LAILAPS for ‘low nitrogen in maize’ results in the return of nine proteins that are referenced by 13 unique OPTIMAS unigenes, with two of those unigenes (OptiV1C17314 and OptiV1S22914) significantly down-regulated under low nitrogen conditions. While the down-regulation of OptiV1C17314 has been experimentally validated (Schlüter et al. 2013), the down-regulation of OptiV1S22914 (annotated to UniProt accession No. B1P123 encoded by the genes BX7/ZRP4) is only predicted by LAILAPS, as this gene possesses a similar expression profile to that of OptiV1C17314. Both are expressed in seedlings and newly formed maize crown roots. Its role in nutritional deficiency is not finally investigated. Additionally, the top-ranked unigene link is linked to UniProt accession No. Q84VI9 which has a link to OPTIMAS unigene OptiV1C15609. Q84VI9 corresponds to the gene ZmNrt2.1, which encodes a putative high affinity nitrate transporter. Quaggiotti et al. (2003) proved that the gene ZmNrt2.1 plays a role in the maize stress response to low nitrogen. Follow-up analyses of these 13 unigenes should consider metabolic, regulatory and sequence properties to characterize their role fully in plant metabolism under low nitrogen conditions, but such analyses are beyond the scope of this paper.

## Discussion

LAILAPS offers a new level of comprehensive information retrieval in plant research. It is designed to guide biologists with different levels of expertise and backgrounds to their data of interest by allowing a broad and deep search of a number of plant genome databases. The wide spectra of information domains, cross-references and data structures in LAILAPS support a maximum use of information potential from distributed and heterogeneous plant genome resources. LAILAPS is able to satisfy individual information demands using a feedback system and evidence-filtered merging of cross-referenced data. This ranges from specific investigations of particular biological entities such as genes, to the search for particular traits or metabolic functions, to broad scans of available genomic knowledge about specific taxonomies.

As discussed (see Table 2), LAILAPS’s user pertinence and query assistance are superior to those of other commonly used IR systems. Regarding the amount of expected query results, LAILAPS is similar to established systems such as UniProt and NCBI GQuery. For a query term, UniProt ranks results similarly to LAILAPS, and top-ranked results in LAILAPS are also found among the top-ranked UniProt results. However, strictly counting the number of results can be misleading in respect to the potential information density. UniProt results can include frequently replicated data (e.g. TrEMBL records of computationally predicted annotations), and the first result page produced via a GQuery search is simply an overview of the hit number in NCBI-indexed databases. Because GQuery does not merge the hits together into one ranking system, the user is forced to decide which special data source is the most interesting, which is a time-consuming process.

We demonstrated that in comparison with the Apache Lucene information retrieval API (application programming interface), which is one of the most widely used relevance scoring systems in life science information systems, LAILAPS demonstrates an increased discriminating performance. This is because while Lucene represents documents and queries as weighted vectors in a VSM, where each distinct index term is a vector dimension and weights are TF–IDF values, LAILAPS scores documents in relation to the query terms as an 11-dimensional feature vector and estimates the relevance using a neural network.

Finally, we validated LAILAPS performance and demonstrated its utility in knowledge discovery cases through two examples. In the first, LAILAPS was able to identify accurately an already validated gene of interest, notably even though two different, yet closely related, query terms were used. In the second example, the linked genome information provided through the LAILAPS query provided information about already validated genes involved in a particular process of interest but also predicted the involvement of other genes in the process, which the researcher can now fully characterize and validate.

## Materials and Methods

**Fig. 3:**
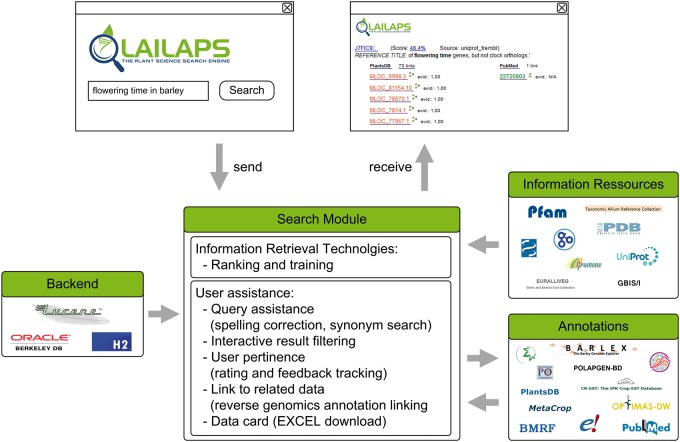
Overview of the LAILAPS architecture and workflow. On the client side (web browser), the user makes a request, which is sent to the server. Information resources and annotations are stored using different backend systems. Processing and search modules are used to find documents that are related to a request. All results are received by the client and can be investigated on the web browser or downloaded for later investigation.

LAILAPS is based on a client–server architecture. Users send requests, which are received and then processed by the server. The results, in the form of a list of ranked documents with linked genomic data, are delivered back to the client. Different technologies and algorithms are used in the backend to process the request and analyze the data, and are illustrated in Fig. 3.

### Data management

Data are either stored in LAILAPS as quarterly updated information or linked to another system as an annotation. The storage backend is divided into three parts. Two databases store information while the software library Apache Lucene creates indices to allow a fast information search. Data located in the H2 database (http://www.h2database.com) are accessed during more complex SQL queries, while data stored in the Oracle Berkeley DB (http://www.oracle.com/us/products/database/berkeley-db) are used for key queries. Mapping files link genes to functional annotations. The sources are from genome annotation projects, provided by the transPLANT project partners and further genome consortia. They will be updated on demand and comprise tables of gene IDs and references to fact databases, such as protein knowledge resources, ontologies of gene functions and literature repositories. If a link between indexed facts and genomic data is included in one of the mapping files and the particular fact is relevant for a search query, all related genes appear as links.

### Results ranking

Search modules support IR technologies and user assistance methods for information extraction and ranking, providing a personalized results ranking for the user. A special feature-ranking model, which recognizes features such as attribute, database, keyword, frequency and co-occurrence of queries, organism, sequence length, text position and synonyms, was created in LAILAPS (Lange et al. 2010; and see Table 4 for a list and description of these features). The ranking system is a central component of the search module and is responsible for matching queries with indexed documents. The features are ranked by an artificial neural network, which is a machine learning method that learns from trained data and predicts document relevance. The neural network predicts a vector of 11 relevance feature values obtained from database entries. A feed-forward neural network with 11 neurons at the input and 16–8 neuron architecture in the hidden layer is used to train the data and the neurons are activated with a sigmoid function. The ranked results and all influencing features for each document are presented as additional information and can be inspected by the user (see Fig. 1, part 3—Score link).

**Table 4:** Neural network features and feature descriptions

Feature	Description
Attribute	Attribute for which the query term was found
Database	Database in which the database entry is included
Frequency	Frequency of all query terms in the database entry and attribute
Co-occurrence	Closeness and order to the document terms
Keyword	Provides information regarding whether good or bad keywords are present near the query terms
Organism	Organism the database entry relates to
Sequence length	Length of the sequence described by the database entry
Text position	Portion of the attribute that is covered by the query term
Synonym	Provides information regarding whether the hit was produced by an automatic synonym expansion

### Gene annotations

Gene annotations are sorted by their evidence values. BMRF-linked annotations get ranked by their special gene predictions, which are described in Bargsten et al. (2014). Barley genes are divided into high- and low-confidence genes, as described in International Barley Sequencing Consortium (2012). PlantsDB and BARLEX (barlex.barleysequence.org) both implement this basic evidence classification for high- and low-confidence values. If no evidence is provided, the annotations are ordered by identifier and marked in LAILAPS with ‘N/A’.

## Funding

This work was supported by the European Commission [within its 7th Framework Program, under the thematic area Infrastructures (contract No. 283496) and carried out under the framework of the transPLANT project].

## Abbreviations

API: application programming interface
GO: Gene Ontology
IR: information retrieval
TF–IDF: term frequency–inverse document frequency
VSM: vector space model
